# Point Prevalence Survey of Antimicrobial Utilization in Ghana’s Premier Hospital: Implications for Antimicrobial Stewardship

**DOI:** 10.3390/antibiotics10121528

**Published:** 2021-12-14

**Authors:** Daniel Ankrah, Helena Owusu, Asiwome Aggor, Anthony Osei, Agneta Ampomah, Mark Harrison, Frempomaa Nelson, Grace Owusu Aboagye, Priscilla Ekpale, Jennifer Laryea, Julia Selby, Serwaa Amoah, Linda Lartey, Okaikor Addison, Elizabeth Bruce, Joyce Mahungu, Mariyam Mirfenderesky

**Affiliations:** 1 Korle-Bu Teaching Hospital, Accra GA-221-1570, Ghana; h.owusu@kbth.gov.gh (H.O.); Waggor23@yahoo.com (A.A.); tonyosei3@gmail.com (A.O.); agneta_ampomah@yahoo.com (A.A.); markharry02@yahoo.com (M.H.); f.nelson@kbth.gov.gh (F.N.); g.aboagye@kbth.gov.gh (G.O.A.); priscillaekpale@gmail.com (P.E.); jencoke@hotmail.com (J.L.); julinat@yahoo.com (J.S.); s.amoah02@kbth.gov.gh (S.A.); linlartey@gmail.com (L.L.); o.addison@kbth.gov.gh (O.A.); lizzybruce59@yahoo.com (E.B.); 2 North Middlesex University Hospital NHS Trust, London N18 1QX, UK; joyce.mahungu@nhs.net (J.M.); mariyam.mirfenderesky@nhs.net (M.M.)

**Keywords:** point prevalence survey, CwPAMS, antimicrobial stewardship, Korle Bu Teaching Hospital, Ghana

## Abstract

The first comprehensive point prevalence survey at the Korle Bu Teaching Hospital (KBTH) was performed as part of the 2019 Global Point Prevalence Survey (Global-PPS) on antimicrobials. The aim was to establish a PPS baseline for the whole hospital and to identify required stewardship interventions. The PPS was conducted over three days in June 2019 using the GLOBAL-PPS standardized method for surveillance of antimicrobial utilization in hospitals to evaluate antimicrobial prescribing. In all, 988 patients were admitted to 69 wards. Overall antimicrobial prevalence was 53.3%. More community-acquired infections (CAI) were treated empirically compared to health-care associated infections (94.0% vs. 86.1% respectively, *p* = 0.002). Main indications for prescribing antimicrobials were pneumonia (18.4%), skin and soft tissue infections (11.4%) and sepsis (11.1%). Among antimicrobials, systemic antibiotics accounted for 83.5%, of which amoxicillin with beta-lactam inhibitor (17.5%), metronidazole (11.8%) and ceftriaxone (11.5%) dominated. Guideline compliance was 89.0%. Stop/review dates were completed in 33.4% and documented reason was recorded in 53.0% of all prescriptions. If the findings in this PPS can be addressed antimicrobial stewardship at the KBTH stands to improve significantly.

## 1. Introduction

Resistance to antimicrobial medicines and the resultant loss of their effectiveness and treatment failure has become a frightening global health problem [1]. Resistant infections lead to substantial economic burden, morbidity and mortality [2,3]. Resistant infections were responsible for 25,000 deaths per year with a cost of EUR 1.5 billion to the health system in Europe [3]. When resistance to first-line antimicrobial drugs occurs, more expensive therapies may be used, and longer duration of treatment and hospitalized care are usually required [2]. Globally, there is significant resistance to both old and new anti-microbial drugs, including third generation cephalosporins, carbepenems and fluoroquinolones [4,5], yielding a phenomenon of hard-to-treat infections.

In sub-Saharan Africa, paucity of evidence on antimicrobial resistance (AMR) reflects an underestimated magnitude of the problem, but in countries where data is available, substantial AMR has been found with a rate of 100 percent among some bacteria [6,7]. Resistance to third generation cephalosporins, fluoroquinolones, penicillins, chloramphenicol, nalidixic acid and co-trimoxazole in sub-Saharan Africa [8,9,10,11] has created a challenge for the treatment of infections.

Several factors contribute to antimicrobial resistance. These include inappropriate antimicrobial use and lack of surveillance systems contributing significantly to the spread of antimicrobial resistance. Poor infection prevention and control in healthcare facilities, lack of available, affordable and rapid diagnostic tests, and low-quality medicines are other factors that influence antimicrobial resistance [2,12]. Disparities in proper use of antibiotics, infection treatment and hygiene practices, infectious disease burden and availability of first- and second-line drugs contribute to geographical differences in AMR [12,13].

Antimicrobial consumption contributes to AMR, and the volume of antimicrobial consumption influences resistance. Global antibiotic consumption has grown in recent years with low- and middle-income countries having the highest increase [14]. Between 2000 and 2010 antibiotic, use rose by 30%, and the rise in consumption for the treatment of infections such as pneumonia is expected to continue. Heavy antibiotic use by hospitals generates some of the most dangerous and difficult-to-treat infections [15]. Globally, one-third of hospitalized patients receive at least one antimicrobial prescription, and about 90% of antimicrobials prescribed are systemic antibiotics [16]. Penicillins with β-lactamase inhibitors, third-generation cephalosporins and fluoroquinolones are the most frequently prescribed antibiotics for hospitalised patients [16]. Only one-fifth of antimicrobial medicines prescribed for hospitalized patients target a specific micro-organism [16]. Recent data show that a significant proportion of prescriptions do not specify duration of treatment and reason for use [16]. However, significant variations exist in antimicrobial use between regions across the globe. Africa is the region with the highest antimicrobial use, predominantly for community acquired infections. Antimicrobial medicines are prescribed for half of hospitalized patients in Africa, and in some countries, antimicrobial use is as high as 75 percent [16]. Further, Africa has the highest use of antimicrobial drugs without reason and the lowest targeted use for resistant organisms [16]. Ghana, similar to many sub-Saharan African countries, has limited evidence on antimicrobial drug use in hospitalized patients.

In Ghana, AMR is prevalent, with rates exceeding 75 percent by some organisms [17]. Methicillin resistant staphylococcus aureus (MRSA), streptococci, salmonella, and *E. coli* have demonstrated high resistance to antibiotics in Ghana [17]. Low susceptibility of bacteria to antibiotics such as tetracycline, co-trimoxazole, nalidixic acid and some penicillins, and emerging resistance to quinolones, cephalosporins, gentamycin and carbapenems in Ghana [17,18] is threatening morbidity and mortality outcomes of infectious diseases.

To provide feedback on antimicrobial use and associated resistance and to assess the effect of interventions and improve antimicrobial decision making, surveillance systems must be implemented as part of antimicrobial stewardship programmes [19,20,21]. Surveillance as part of stewardship programmes improves stewardship interventions such as promoting guideline adherence in empirical treatment [22,23]. Collecting hospital antimicrobial data and implementing informed interventions for optimal antibiotic use in hospitals has significant potential to reduce antimicrobial resistance at local, national, regional and global levels.

Antibiotic use data collection methods in hospitals allow standardization and comparison of antimicrobial use between hospitals, districts, countries, and regions. A typical example is the antimicrobial point prevalence survey (PPS). The PPS enables data collection with minimized workload and resource requirements at a specific time point, and it is already in use in hospitals [24]. The Global Point Prevalence Survey (Global-PPS) of antimicrobial drug use and resistance is suited for resource-limited (low- and middle-income) countries and allows comparison of data with high-income countries.

Similar to many sub-Saharan African countries, limited evidence on antimicrobial use with standardized surveillance methods in Ghanaian hospitals stifles the provision of timely and efficient feedback to the health system, and adversely affects the development of evidence-based local antimicrobial stewardship programmes and other interventions aimed at reducing antimicrobial resistance. This study, which is the first comprehensive antimicrobial survey at the Korle Bu Teaching Hospital (KBTH), used the PPS method and was a partnership between Korle-Bu Teaching Hospital from Ghana and the North Middlesex University Hospital (NMUH) from the UK. It was part of the 2019 Global Point Prevalence Survey (Global-PPS) on antimicrobials.

## 2. Results

### 2.1. Antimicrobial Prevalence

Over the course of 3 days, 988 patients on 69 wards were surveyed, and 527 patients received a total of 967 antimicrobials, giving an overall antimicrobial prevalence of 53.3% (Table 1). One hundred and eighty-two patients (35%) received one antimicrobial, 263 patients (50%) received two antimicrobials, and 82 patients (15%) received three or more antimicrobials.

After the intensive care wards, the paediatric medical wards had the highest antimicrobial prevalence at 76.3% (Table 1). The majority of patients received antimicrobials via the intravenous route of administration (62.5%).

### 2.2. Patient Demographics

Of the 527 patients treated with antimicrobials, 72% were adults (≥18 years), and 28% were children or neonates, of which 56% were 24 months or younger (Table 2). The total number of children exceeds that on the dedicated paediatrics wards, as children may be based on adult wards, usually housed with adult females, particularly on specialist units.

Sixty-two percent of treated adult inpatients were female, with an average age of 38 years compared with 48 years for males. The hospital has a large obstetric service with a head count of 229 at the time of the survey, of which 110 were receiving at least one antimicrobial, accounting for 29% of the total antimicrobial consumption in the adult population.

### 2.3. Treatment Indications

#### 2.3.1. Community-Acquired versus Healthcare Associated Infections

Forty-one percent (41%) and 15% of antimicrobials were prescribed for community-acquired (CAI) and healthcare-associated infections (HAI), respectively. Of these, 6% of CAI, versus 13.9% of HAI (*p* = 0.002), were targeted therapies (Table 3).

#### 2.3.2. Prophylactic Antimicrobials

Thirty-six percent of all prescribed antimicrobials (*n* = 967) were for prophylactic use, of which 91.4% (*n* = 318) were used for surgical prophylaxis. One hundred and eighty-four patients (out of the 527 surveyed) were prescribed antimicrobials for surgical prophylaxis, of which 78.0%, 13.0% and 9.0% received treatment for more than one day, one day and a single dose, respectively.

The use of prophylactic agents in obstetric and gynaecological surgery comprised 15% of overall prescriptions. One hundred and ten out of 229 (48.0%) obstetric patients were prescribed antimicrobial therapy, of which 75% was for prophylaxis post-surgery, with the majority of prescriptions lasting more than one day (64%).

#### 2.3.3. Most Common Diagnoses Treated with Therapeutic Antimicrobials

The most common diagnosis requiring therapeutic antimicrobials was pneumonia, followed by treatment for skin and soft tissue infections (Table 4) and sepsis.

### 2.4. Antibiotic Quality Indicators by Activity

Documentation of diagnosis/indication in the patients notes ranged from 76.1% in medicine, to 41.2% in ICU **(**Table 4). Whilst antimicrobials were compliant with guidelines in 80–90% of cases, lack of guidelines was documented in all units, with almost one-third of surgical prescriptions written without a current guideline. Prescriptions had a current stop or review date in less than 50% of cases across all activities and was lowest in the ICU at 11.8%.

### 2.5. Antibacterial Resistance

Forty-five patients had targeted therapy, with multi-drug resistance detected in 57.8% of isolates, comprising 4.9% of all treated inpatients (Table 4).

### 2.6. Antimicrobial Class

Nine hundred and sixty-seven (967) individual prescriptions were recorded, with 83.5% comprising antibacterials for systemic use (Table 5). Accounting for 18.3% (177/967) of prescriptions, metronidazole was the single most common antibiotic prescribed (ATC codes J01XD and P01AB). Antimicrobials in amount of 3.0%, 2.1%, 1.1% and 1.0% were prescribed as treatments for malaria, HIV/AIDS, fungal disease (triazole derivatives), and Mycobacterium tuberculosis, respectively.

#### 2.6.1. Antibacterials for Systemic Use (Therapeutic and Prophylactic)

After metronidazole (J01XD and P01AB), penicillins with beta-lactamase inhibitors were the most common antibiotic for systemic use and were prescribed followed by second and third generation cephalosporins, fluoroquinolones, beta-lactamase resistant penicillins and aminoglycosides (Table 6).

#### 2.6.2. Proportional Antibiotic Use by Class on Ward

**Figure 1:**
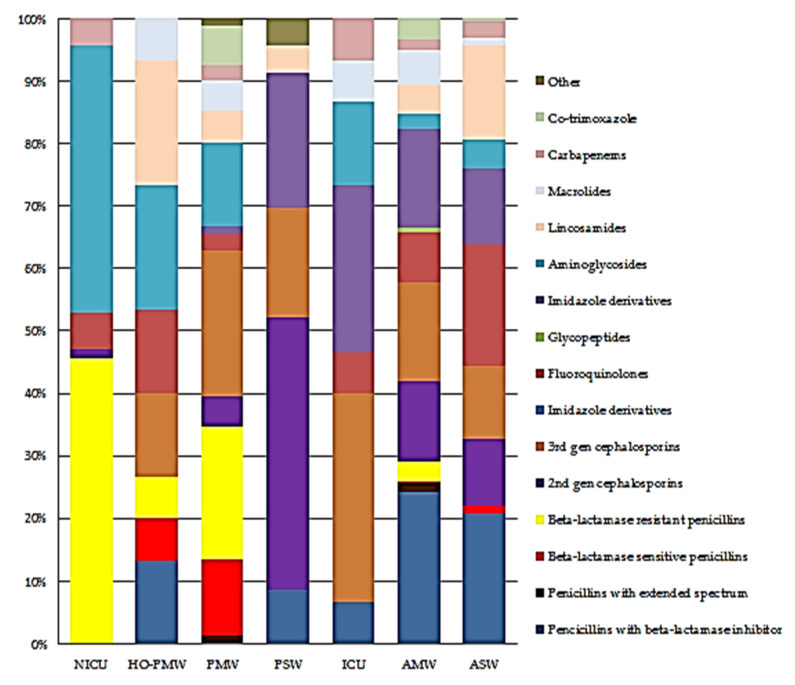
Proportional antibiotic use (J01) by class on wards. Abbreviations: NICU: neonatal intensive care unit, HO-PMW: haematology–oncology–paediatric medical ward, PMW: paediatric medical ward, PSW: paediatric surgical ward, ICU: intensive care unit, AMW: adult medical ward, ASW: adult surgical ward.

**Figure 2:**
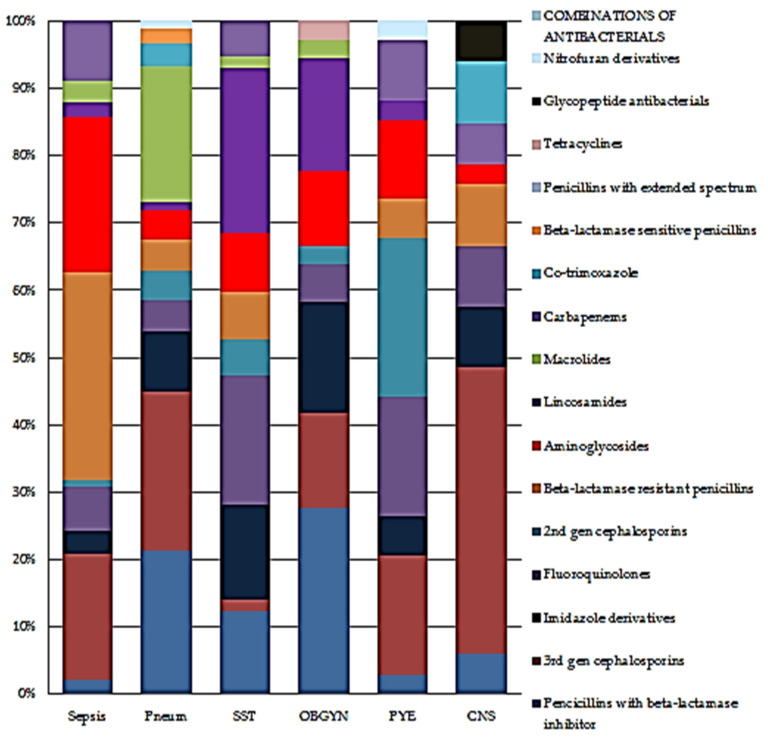
Top 6 indications disaggregated by antibiotic class. Abbreviations: Pneu: pneumonia or lower respiratory tract infection, SST: skin and soft tissue, OBGY: obstetric/gynaecological infections, Pye: upper urinary tract infection, CNS: infection of central nervous system.

Proportional antibiotic use by class on the neonatal, paediatric, adult medical and surgical wards, and the top antibiotic indications disaggregated according to class are shown in Figure 1 and Figure 2.

## 3. Discussion

This was a comprehensive antimicrobial PPS that involved all clinical departments, wards and units of the KBTH. Data collection was carried out over a 3-day period, and all patients’ folders were assessed. Data were collected on those patients on at least one antimicrobial agent during the survey period.

### 3.1. Antimicrobial Prevalence

This survey identified an overall prevalence of antimicrobial prescriptions among inpatients at the Korle Bu Teaching Hospital to be 53.3%. Among the inpatients surveyed, 83.5% of them received systemic antimicrobials and 65% received more than one antimicrobial. The prevalence rate in this study is comparable to previous studies performed in KBTH among inpatients which found prevalence rates of 51.4% in 2006 [25] and 53% in 2000 [26]. This shows that rates of antibiotic use among inpatients in KBTH have been fairly consistent over the past two decades. Studies from other hospitals in Ghana showed comparatively higher prevalence rates with Komfo Anokye Teaching Hospital reporting a prevalence of 64% [27]; Ho Teaching Hospital recorded 66.7% [28], and Keta Municipal Hospital recorded 82.0% [29]. Prevalence from this study compared favourably with antimicrobial prevalence in Africa (63%) but is still relatively high compared to North America (34.0%), South Europe (31.7%) or East Europe (30.3%) [16]. Higher rates of antimicrobial use are typically linked with the development of antimicrobial resistance and healthcare-associated infections [30] and thus must be suitably justified.

### 3.2. Most Common Antimicrobials Prescribed

The top four antibiotics prescribed, metronidazole, amoxicillin/clavulanic acid, ceftriaxone and cefuroxime, reinforce the work of Labi et al. [25] which reported the same antibiotics as the four most used antibiotics at the Korle Bu Teaching Hospital. Beta-lactam antibiotics (cephalosporins and penicillins) were the most frequently used classes. Fluoroquinolones were more frequently prescribed than macrolides and aminoglycosides. The antibiotic use pattern found in this study is largely consistent with those of recently published point prevalence survey of antibiotics [16,31,32,33] These results suggest that beta lactam antibiotics remain drugs of choice in the management of infections. The usefulness of this group of antibiotics in the future is however threatened, as data show that increased antibiotic use is associated with antimicrobial resistance [34,35,36,37,38,39].

### 3.3. Most Common Diagnosis for Antimicrobial Use

The top three morbidities requiring antimicrobial therapy in the hospital were pneumonia, skin and soft tissue infections and sepsis with upper respiratory tract infections and malaria as the fourth and fifth topmost respectively. The results are similar to a study carried out in a referral hospital in Kenya in which the respiratory system had the largest proportion of antibiotics prescribed followed by skin, soft tissue, bone and joint (SSTBJ) infections [32]. It is also quite similar to a survey carried out in 18 hospitals in Egypt, in which amongst patients receiving antibiotics for treatment of infection, the most common anatomical sites of suspected or proven infection were the respiratory tract, gastrointestinal tract, and skin, bone and joints [40].

Further, in a survey involving paediatric patients from 26 Canadian hospitals, the most commonly treated infections were sepsis (16%) and lower respiratory tract infection (12.1%) [41]. The consistency in these results may justify the prescribing of these medicines; however, care should be taken to avoid over prescribing.

### 3.4. Targeted versus Empiric Treatment

Most treatments were empirical for both healthcare- and community-acquired infections. This finding is consistent with recent data from Africa [42] and other developing countries [43,44]. Empirical treatment for community-acquired infections had a higher proportion compared to empirical treatment for healthcare-acquired infections. Versporten et al. published similar findings [16]. Empirical treatment of infections can be attributed to limited laboratory resources to aid in the identification of specific pathogens and the antimicrobials they are most susceptible to. This results in the use of broad-spectrum empiric treatment in a bid to cover all possible pathogens that could be responsible for the infection. In a randomized comparative prospective study of complications in transrectal prostate biopsy [44] post-prophylactic treatment, it was found that infection rates were significantly lower in targeted treatment compared to empirical treatment. Further, the supremacy of targeted treatment over empirical treatment was manifested in a meta-analysis [45]. The increased empirical treatment of infections in the hospital may contribute to the burden of a microorganism. While it has been reported that tertiary hospitals in Ghana perform better in targeted treatment of infections compared to non-tertiary (secondary) settings [29], results from this study implies that targeted treatment needs more improvement. The hospital’s Drugs and Therapeutics Committee may lead the way in this regard.

### 3.5. Quality Indicators for Prescribing

In this era of increasing AMR, policies that seek to promote prudent antimicrobial prescribing have been developed and adopted by countries and hospitals [46]. An evidence-based antimicrobial guideline is preferably the mainstay of such policies [47]. Adherence to such hospital guidelines is however often low to moderate [48,49]. Korle bu Teaching hospital had some positive practices regarding antimicrobial stewardship among the indicators evaluated, but this varied widely by ward type. Guideline compliance was greater than 80% among all the wards surveyed, and this was quite laudable. However, when it came to documenting the reasons for antimicrobial prescriptions, the medical wards fared much better than the surgical or ICU (76.1% vs. 44.3% vs. 41.2% respectively). Possible reasons for these results could be the focus of future studies.

Another important indicator for prudent antimicrobial prescribing is the presence of a stop or review date. Here, the ICU fell short compared to the surgical and medical wards (11.8% vs. 49.9% vs. 38.5%, respectively). A possible reason for this could be the daily review of patients on the ICU wards, hence prescribers not appreciating the need to indicate the stop or review date. An intervention targeting ICU prescribers to include this parameter in their notes will be important for KBTH to improve antimicrobial stewardship.

Considering biomarkers, patients pay per laboratory tests that are not reimbursed; hence, c-reactive protein (CRP) and procalcitonin are often viewed as non-essential expensive extras.

Point prevalence surveys are among the most reliable tools in the assessment of antibiotic utilization at the patient level [50]. They are also valuable in providing information on antimicrobial resistance. Access to antimicrobial utilization and resistance patterns are pivotal for the success of any antimicrobial stewardship (AMS) programme. In a systematic review and meta-analysis, a significant association was reported between an AMS programme and a decrease in the incidence of antibiotic infections among hospitalized patients [51]. This calls for regular PPS at all health institutions with the aim of identifying the gaps and fixing them to improve public health.

### 3.6. Limitations

Data collection in this study was performed over three days only. The seasonal nature of some diseases (asthma, flu, etc.) that require the use of antimicrobial agents may change the prevalence of figures depending on the time the study is performed. This calls for continuous PPS at different times in the year before the actual antimicrobial prevalence in the hospital can be determined. For Ghana, there are only two clear seasons (rainy season and dry season), and the effect may not be as significant compared to countries with four seasons. It is possible that reported figures for microbiological reports may be less than actual because these may have been absent during the survey period, most probably due to the time it takes for such reports to be ready. We did not report on the number of various laboratory tests (blood culture, stools for *C. difficile*, etc.) performed per year, to avoid under ascertainment. This is because some of the requested tests were performed in private labs outside the hospital.

## 4. Materials and Methods

### 4.1. Study Design

A cross-sectional study design was used to investigate antimicrobial use and resistance among all inpatients at the Korle Bu Teaching Hospital from the 19–21 June 2019. The primary outcome was to understand antimicrobial prescribing rates, antimicrobial indications and agent selection. The secondary outcome was to look at key prescribing indicators and resistance rates, if available.

#### 4.1.1. Study Site

The point prevalence survey was based at the Korle Bu Teaching Hospital, Accra. The Korle Bu is a teaching hospital affiliated with the University of Ghana Medical School. It is currently the third largest Hospital in Africa and the leading national referral centre for Ghana and beyond, including the National Reconstructive Plastic Surgery and Burn Centre, the National Cardiothoracic Centre and the National Centre for Radiotherapy and Nuclear Medicine.

The Hospital has a 2000-bed capacity, with an average daily attendance of 1500 outpatients and approximately 250 patient admissions. There are 17 clinical and diagnostic departments/units including all branches of medicine and surgery, including neurosurgery, radiotherapy and oncology, paediatric surgery, dentistry, psychiatry, ophthalmology, child health and obstetrics and gynaecology.

#### 4.1.2. Pre-Survey

Eleven pharmacists and 8 intern pharmacists underwent a 2-day training workshop in June 2019, covering the foundations of antimicrobial surveillance and the global-PPS methodology [31]. The training was conducted by a consultant microbiologist and an antimicrobial pharmacist from the North Middlesex University Hospital, London, UK.

#### 4.1.3. Study Population

All patients on all wards in the hospital admitted at 08:00 h on the day of the survey formed the baseline population of the survey (denominator).

#### 4.1.4. Sampling Method

Patient folders and medication charts were screened for eligibility. For inpatients receiving at least one antimicrobial, detailed information was collected using standardized data collection forms (http://www.global-pps.com, accessed on 1 June 2019).

#### 4.1.5. Inclusion Criteria

Inclusion criteria as per the global-PPS: all inpatients on any antibiotic before 8 am on the day of the survey; patients hospitalized as an inpatient at or before 08:00 h; antibiotic administered orally, parenterally, rectally or through inhalation; ongoing treatment at 08:00 h. In addition, for surgical patients, the dosage and time of administration of prophylactic antimicrobials before or after surgery were obtained to determine the duration and frequency of prophylaxis.

#### 4.1.6. Exclusion Criteria

Exclusion criteria as per the global-PPS: hospitalized after 08:00 h, outpatient clinic, day surgery/day treatment, emergency room, outpatient dialysis, discharged patients waiting for transportation, parents/relatives of admitted children, outpatient parenteral antibiotic therapy (OPAT), topical antibiotics, ophthalmologic antibiotics, treatment initiated after 08:00 h, treatment discontinued before 08:00 h.

### 4.2. Data Collection

Pharmacists and interns were divided into groups and dispatched to departments. Ward level data was captured, including information on the type of ward, number of eligible patients, and characteristics of the ward. Patient level data included information on the antimicrobial agents prescribed, indication, laboratory data, stop/review date and other quality indicators. Guideline compliance was according to the National Ghana treatment guidelines or local treatment guidelines if applicable. For this study, the Standard Treatment Guideline of the Ghana National Drugs Programme [52] (GNDP) as well as the local guideline at the department of obstetrics and gynaecology were used. All data collection forms were centrally validated to ensure consistency across groups. Using the 5 moments of hand hygiene, train-the-trainer sessions on infection prevention and control (IPC) were organized, followed by hand hygiene audits and instant feedback. The report on IPC is being handled separately from the PPS report.

### 4.3. Data Entry

Collected data were entered into a web-based tool for data entry and validation designed by the University of Antwerp (http://global-pps.com, accessed on 1 June 2019) by two data analysts with oversight from the United Kingdom partners.

### 4.4. Data Analysis

Validated data were exported to Microsoft Excel and analysed with Microsoft Excel 2016. We examined antimicrobial utilization and assessed percentage adherence/compliance to health policy guidelines. Further, we assessed levels of targeted and empirical treatment against health care associated infections (HAI) defined as “infection detected within 48 h of hospital admission in patients that had previous contact with healthcare service within one year”, and community acquired infections (CAI), defines as “infection detected within 48 h of hospital admission in patients without previous contact with healthcare service” [53]. In addition, we ascertained morbidities that were treated using antimicrobial agents. These were based on agreed quality indicators from the GPPSS instrument used (See Supplementary Material S1).

### 4.5. Ethical Issues

Ethical approval was not sought, as this was a quality improvement project that needs to be performed continuously.

## Figures and Tables

**Table 1:** Antimicrobial prevalence by ward type.

Ward	% Treated (Number Treated)
General or mixed Adult ICU	100.0 (7)
General or mixed Adult MW	53.6 (246)
General or mixed Adult SW	50.0 (162)
Haematology-Oncology PMW	47.6 (10)
Neonatal Intensive Care Unit	47.7 (41)
Paediatric MW	76.3 (45)
Paediatric SW	50.0 (16)
Antimicrobial prevalence	53.3 (527)

Abbreviations: ICU: intensive care unit, MW: medical ward, SW: surgical ward, PMW: paediatric medical ward).

**Table 2:** Demographics of treated patients.

Patients					Total 527
Adults ≥ 18 years				379	(72%)
	Female	Male	Unknown		
≥60 years	210	104	1	315	(83%)
>60 years	25	39		64	(17%)
Median age in yrs (iqr *)	34 (16)	45 (29)			
Children < 18 years				148	(28%)
	Female	Male	Unknown		
<18 years	18	32		50	(34%)
<5 years	8	7		15	(10%)
<24 months	41	41	1	83	(56%)
Median age in years (iqr)	0.3 (7)	1.5 (8)			

* interquartile range.

**Table 3:** The proportion of antimicrobials for empirical versus targeted treatment per therapeutic use (CAI or HAI).

	Empirical		Targeted		Total	
	N	%	N	%	N	%
CAI	374	94.0	24	6.0	398	73.4
HAI	124	86.1	20	13.9	144	26.6
	498		44		542	

Abbreviations: CAI: community-acquired infection, HAI: healthcare-associated infection.

**Table 4:** Various characteristics of the PPS.

Characteristic	Number	(%)
Ten commonest diagnoses treated with antimicrobials		
Pneumonia	58	18.4
Skin and soft tissue	36	11.4
Sepsis	35	11.1
Upper respiratory tract infection	25	7.9
Malaria	24	7.6
Infection of central nervous system	22	7.0
Obstetrics/gynaecology infection	22	7.0
Bone and joint infection	15	4.8
Gastro-intestinal infection	14	4.4
Intra-abdominal sepsis	10	3.2
Treatment according to biomarkers (*n* = 527)		
No	502	95.3
Yes	25	4.7
Antibiotic quality indicators by department		
Medical		
Reason in notes	235	76.1
Guidelines missing	79	25.6
Guideline compliant	122	85.9
Stop/review date	119	38.5
Surgical		
Reason in notes	183	44.3
Guidelines missing	127	30.8
Guideline compliant	135	83.3
Stop/review date	206	49.9
Intensive care unit		
Reason in notes	35	41.2
Guidelines missing	11	12.9
Guideline compliant	38	92.7
Stop/review date	10	11.8
Patients with isolated multi-drug resistant pathogen		
Third generation cephalosporin resistant (TGCR)	2	4.4
Carbapenem-resistant enterobacteriaceae	2	4.4
Carbapenem-resistant non fermentor Gram-negative bacilli	2	4.4
ESBL-producing Enterobacteriaceae	7	15.6
ESBL-producing Enterobacteriaceae-TGCR	1	2.2
No MDR recorded	19	42.2
Targeted treatment against other MDR organisms	10	22.2
Vancomycin-resistant enterobacteriaceae (VRE)	2	4.4

**Table 5:** Total systemic antimicrobials prescribed by class (WHO ATC classification, 2020).

ATC Code	Total Systemic Antimicrobials	No (%)	
J01	Antibacterials for systemic use	807	83.5%
P01AB	Nitroimidazole derivatives	83	8.6%
P01B	Antimalarials	29	3.0%
	P01B	28	
	P01BB	1	
J02	Antimycotics for systemic use	10	1.0%
	J02AC		
J04	Antimycobacterials	10	1.0%
	J04AM		
J05	Antivirals for systemic use	20	2.1%
	J05AR	9	
	J05AF	6	
	J05AG	4	
	J05AB	1	
A07AA	Intestinal Anti-infectives	7	0.7%
D01	Antifungals for dermatological use	1	0.1%
	D01BA		
		967	100%

P01B: artemisinin and derivatives including combinations, P01BB: biguanides, J02AC: triazole derivatives, J04AM: combinations of drugs for treatment of tuberculosis, J05AR: antivirals for treatment of HIV infections, combinations, J05AF: nucleoside and nucleotide reverse transcriptase inhibitors, J05AG: non-nucleoside reverse transcriptase inhibitors, J05AB: nucleosides and nucleotides excl. reverse transcriptase inhibitors.

**Table 6:** Antibacterials for Systemic Use (Therapeutic and Prophylactic).

Therapeutic Prescription	No
Penicillins with beta-lactamase inhibitor	141
3rd gen cephalosporins	113
2nd gen cephalosporins	86
Beta-lactamase resistant penicillins	67
Beta-lactamase sensitive penicillins	12
Penicillins with extended spectrum	8
Imidazole derivatives	95
Fluoroquinolones	86
Aminoglycosides	66
Lincosamides	62
Macrolides	28
Carbapenems	19
Co-trimoxazole	17
Tetracyclines	3
Glycopeptide antibacterials	2
Combinations of antimicrobials	1
Nitrofuran derivatives	1
	807

## Data Availability

Data from this point prevalent survey are included in the study.

## Supplementary Materials

The following are available online at https://www.mdpi.com/article/10.3390/antibiotics10121528/s1.
